# Autotransporters Drive Biofilm Formation and Autoaggregation in the Diderm Firmicute Veillonella parvula

**DOI:** 10.1128/JB.00461-20

**Published:** 2020-10-08

**Authors:** Nathalie Béchon, Alicia Jiménez-Fernández, Jerzy Witwinowski, Emilie Bierque, Najwa Taib, Thomas Cokelaer, Laurence Ma, Jean-Marc Ghigo, Simonetta Gribaldo, Christophe Beloin

**Affiliations:** a Genetics of Biofilm Laboratory, Institut Pasteur, UMR CNRS2001, Paris, France; b Université de Paris, Sorbonne Paris Cité, Paris, France; c Unit Evolutionary Biology of the Microbial Cell, Institut Pasteur, UMR CNRS2001, Paris, France; d Sorbonne Université, Collège Doctoral, Paris, France; e Hub de Bioinformatique et Biostatistique-Département Biologie Computationnelle, Institut Pasteur, USR 3756 CNRS, Paris, France; f Plate-forme Technologique Biomics-Centre de Ressources et Recherches Technologiques, Institut Pasteur, Paris, France; Princeton University

**Keywords:** *Veillonella*, adhesins, autotransporter proteins, biofilms, diderm firmicutes

## Abstract

The *Negativicutes* are a clade of the *Firmicutes* that have retained the ancestral diderm character and possess an outer membrane. One of the best studied *Negativicutes*, Veillonella parvula, is an anaerobic commensal and opportunistic pathogen inhabiting complex human microbial communities, including the gut and the dental plaque microbiota. Whereas the adhesion and biofilm capacities of V. parvula are expected to be crucial for its maintenance and development in these environments, studies of V. parvula adhesion have been hindered by the lack of efficient genetic tools to perform functional analyses in this bacterium. Here, we took advantage of a recently described naturally transformable V. parvula isolate, SKV38, and adapted tools developed for the closely related *Clostridia* spp. to perform random transposon and targeted mutagenesis to identify V. parvula genes involved in biofilm formation. We show that type V secreted autotransporters, typically found in diderm bacteria, are the main determinants of V. parvula autoaggregation and biofilm formation and compete with each other for binding either to cells or to surfaces, with strong consequences for V. parvula biofilm formation capacity. The identified trimeric autotransporters have an original structure compared to classical autotransporters identified in *Proteobacteria*, with an additional C-terminal domain. We also show that inactivation of the gene coding for a poorly characterized metal-dependent phosphohydrolase HD domain protein conserved in the *Firmicutes* and their closely related diderm phyla inhibits autotransporter-mediated biofilm formation. This study paves the way for further molecular characterization of V. parvula interactions with other bacteria and the host within complex microbiota environments.

**IMPORTANCE** Veillonella parvula is an anaerobic commensal and opportunistic pathogen whose ability to adhere to surfaces or other bacteria and form biofilms is critical for it to inhabit complex human microbial communities such as the gut and oral microbiota. Although the adhesive capacity of V. parvula has been previously described, very little is known about the underlying molecular mechanisms due to a lack of genetically amenable *Veillonella* strains. In this study, we took advantage of a naturally transformable V. parvula isolate and newly adapted genetic tools to identify surface-exposed adhesins called autotransporters as the main molecular determinants of adhesion in this bacterium. This work therefore provides new insights on an important aspect of the V. parvula lifestyle, opening new possibilities for mechanistic studies of the contribution of biofilm formation to the biology of this major commensal of the oral-digestive tract.

## Abstract (precis)

Veillonella parvula is an anaerobic commensal and opportunistic pathogen whose ability to adhere to surfaces or other bacteria and form biofilms is critical for it to inhabit complex human microbial communities such as the gut and oral microbiota. Although the adhesive capacity of V. parvula has been previously described, very little is known about the underlying molecular mechanisms due to a lack of genetically amenable *Veillonella* strains. In this study, we took advantage of a naturally transformable V. parvula isolate and newly adapted genetic tools to identify surface-exposed adhesins called autotransporters as the main molecular determinants of adhesion in this bacterium. This work therefore provides new insights on an important aspect of the V. parvula lifestyle, opening new possibilities for mechanistic studies of the contribution of biofilm formation to the biology of this major commensal of the oral-digestive tract.

## INTRODUCTION

*Negativicutes* are atypical and poorly studied lineages of the *Firmicutes* displaying an outer envelope with lipopolysaccharide (1). Among the *Negativicutes*, *Veillonella* spp. are anaerobic diderm cocci that commonly inhabit the human and animal microbiota. One of their best-studied species, Veillonella parvula (2), is a natural inhabitant of multiple different microbiota, including the human gut (3, 4). V. parvula is considered a commensal organism and is proposed to play a role in the development of immunity through its capacity to colonize the infant gut (5, 6). It is a key early colonizer of the dental plaque during the establishment of sessile microbial communities called biofilms (7), promoting multispecies growth and playing a central role in the metabolism of community members through lactic acid consumption (8). However, V. parvula is also described as an opportunistic pathogen and has been associated with diverse infections, including osteomyelitis, endocarditis, spondylodiscitis, and abscesses as well as systemic infections (9–13).

The importance of V. parvula in the development of the microbial community spurred our interest in identifying the determinants of its adhesion and biofilm formation capacities. Moreover, considering the presence of an outer membrane (OM) in this atypical firmicute, we wondered whether V. parvula uses known diderm or monoderm biofilm determinants or currently undescribed adhesion factors. We recently studied V. parvula DSM2008 as a model diderm firmicute strain (14) to investigate its OM protein composition and detected 78 OM proteins, 13 of which are potential adhesins belonging to the type V family of secreted autotransporter (AT) proteins (T5SS) (15). Autotransporter proteins are specifically found in diderms, and all share common structural and functional features: a Sec-dependent signal peptide, a passenger domain providing the protein function, and an outer membrane β-barrel domain that allows secretion of the passenger domain (16). However, the challenge of genetic manipulation in V. parvula DSM2008 severely limited the study of these adhesins in this strain.

Here, we have sequenced and annotated the genome of V. parvula SKV38, a recently isolated, naturally transformable, and genetically amenable strain (17). We adapted and developed genetic tools for this organism, permitting random and site-directed mutagenesis, plasmid complementation, and controlled expression using an inducible promoter. This enabled us to identify and characterize factors involved in V. parvula biofilm formation. We find that the main V. parvula biofilm-modulating determinants are T5SS adhesins, i.e., typical diderm determinants. Interestingly, the identified adhesins possess an additional C-terminal domain compared to the known domain architecture of classical autotransporters. We also show that a locus encoding a metal-dependent phosphohydrolase HD domain protein is involved in biofilm formation, similarly to what was shown in the prototypical monoderm Bacillus subtilis (18). Therefore, our results demonstrate that diderm firmicutes use a mixture of diderm and monoderm factors to modulate their ability to engage in a biofilm lifestyle, supporting the idea that monoderm and diderm molecular systems could have coevolved in these atypical firmicutes.

## RESULTS

### Random transposon mutagenesis reveals two V. parvula SKV38 genes involved in biofilm formation.

**FIG 1:**
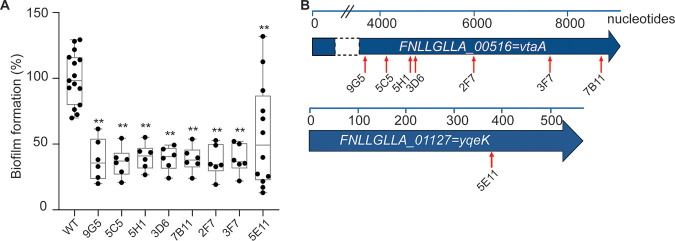
Random transposon mutagenesis in Veillonella parvula SKV38 led to identification of mutants with reduced biofilm formation. (A) Biofilm assay in 96-well polystyrene plates after CV staining of nine transposon mutants identified by random mutagenesis grown for 24 h in BHILC. The mean for the WT is adjusted to 100%. Min-max box plots for 6 or 5 biological replicates for each strain are represented; each replicate is the mean of two technical replicates. *, *P* < 0.05; **, *P* < 0.005 (Mann-Whitney test). (B) Schematic representation of the identified transposon insertion point (red arrow) for the 8 transposon mutants. The blue bar represents the size of the gene in nucleotides.

In order to obtain a framework for genetic work in the recently described naturally competent V. parvula SKV38 isolate, we sequenced it using PacBio technology. We obtained a completely assembled genome of 2.146 Mbp, carrying 1,912 predicted protein-encoding open reading frames (ORFs), 12 rRNAs, 49 tRNAs, and one transfer-messenger RNA (tmRNA) (see Materials and Methods). We performed random transposon mutagenesis in V. parvula SKV38 using the pRPF215 plasmid carrying an inducible transposase and a mariner-based transposon, previously used to mutagenize Clostridioides difficile (19), a close relative of the *Negativicutes*. We screened 940 individual transposon mutants for biofilm formation using crystal violet (CV) staining static biofilm assay in 96-well microtiter plates and identified eight independent mutants with significant reduction in biofilm formation (Fig. 1A). Whole-genome sequencing localized the transposons in two loci putatively implicated in biofilm formation (Fig. 1B). The most affected mutants correspond to insertions in *FNLLGLLA_00516* (seven mutants), encoding a T5SS type Vc trimeric autotransporter. One transposon mutant corresponded to an insertion in *FNLLGLLA_01127*, encoding a putative HD phosphatase (Fig. 1B).

### *FNLLGLLA_00516* encodes a trimeric autotransporter involved in autoaggregation.

**FIG 2:**
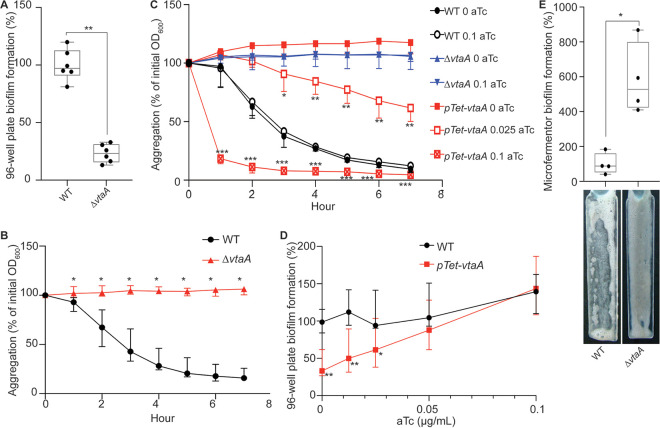
VtaA is an adhesin involved in autoaggregation and biofilm formation. (A) Results of 96-well plate biofilm assay after 24 h growth in BHILC. The mean for the WT is adjusted to 100%. Min-max box plots of 6 biological replicates for each strain are shown. *, *P* < 0.05; **, *P* < 0.005 (Mann-Whitney test between strains). (B and C) Aggregation curves in spectrophotometry cuvette of the WT and *ΔvtaA* strains (B) and of an inducible *vtaA* strain with 0, 0.025, or 0.1 μg/ml of the inducer aTc (C). A value of 100% represent lack of aggregation, and 0% represents complete sedimentation of the culture. Medians from 6 biological replicates are shown, and error bars represent 95% confidence interval. At each time point we performed the Mann-Whitney test between conditions. We applied the Bonferroni correction for multiple testing. Significant *P* values are as follows: *, *P* < 0.004; **, *P* < 0.0004; ***, *P* < 0.00004. The indicated *P* values were calculated by comparing the WT and Δ*vtaA* strains (B) or the P*_tet_-vtaA* strain without aTc and with different aTc concentrations (C). (D) Results of 96-well plate biofilm assay after 24 h of growth of an inducible *vtaA* mutant in BHILC with different concentrations of aTc. The value for the WT without aTc is adjusted to 100%. Medians of 6 biological replicates are shown; each replicate corresponds to the mean of two technical replicates, and error bars represent 95% confidence interval. *, *P* < 0.05; **, *P* < 0.005 (Mann-Whitney test). (E) Biofilm formation in a continuous-flow microfermentor on a glass spatula during 48 h in BHILC. The value for the WT was adjusted to 100%. Min-max box plots of 4 biological replicates for each strain are shown. A picture of the spatula before resuspension is shown below each box plot bar. *, *P* < 0.05 (Mann-Whitney test).

*FNLLGLLA_00516* encodes a protein containing several domains usually identified in the T5SS type Vc trimeric autotransporters. Trimeric autotransporters are OM proteins specific to diderm bacteria that have been widely studied for their ability to bind to different surfaces or to other bacteria (20). *FNLLGLLA_00516* is a homolog of V. parvula DSM2008 *vpar_0464*, which encodes a protein that was detected in the OM (15). *FNLLGLLA_00516* was annotated by PROKKA as BtaF, a trimeric autotransporter identified in Brucella suis involved in adhesion to extracellular matrix and abiotic surfaces (21). Here, we renamed it *Veillonella* trimeric autotransporter A (VtaA), as the first trimeric autotransporter involved in biofilm formation identified in V. parvula SKV38. We deleted the *vtaA* coding sequence and showed that the *ΔvtaA* strain had no growth defect (see Fig. S1A in the supplemental material) but displayed a marked reduction of biofilm formation in 96-well polystyrene microtiter plates (Fig. 2A). Moreover, while V. parvula SKV38 cultures strongly aggregated, the Δ*vtaA* mutant did not (Fig. 2B; see Fig. S2 in the supplemental material). We constructed the P*_tet_-vtaA* strain, where the chromosomal *vtaA* gene is placed under the control of a functional tetracycline/anhydrotetracycline (aTc)-inducible promoter (see Fig. S3 in the supplemental material), and showed that its aggregation capacity and biofilm formation in 96-well polystyrene microtiter plates directly correlated with the aTc concentration (Fig. 2C and D), demonstrating that VtaA-mediated cell-to-cell interactions are critical for biofilm formation under these conditions. Whereas the microtiter plate assay corresponds to a static biofilm assay, we also used continuous-flow glass microfermentors to investigate the contribution of VtaA to biofilm formation under dynamic conditions. Surprisingly, the *ΔvtaA* strain formed almost six times more biofilm than the wild-type (WT) strain under these conditions (Fig. 2E). Accordingly, scanning electronic microscopy (SEM) images of mature biofilms on microscopic plastic slides in a microfermentor showed that the *ΔvtaA* strain formed a much thicker biofilm than the WT (see Fig. S4 in the supplemental material). Altogether, these results suggest that autoaggregation differentially contributes to biofilm formation under static conditions on hydrophobic surfaces versus continuous-flow conditions on hydrophilic surfaces.

### V. parvula SKV38 encodes 16 putative autotransporters in addition to VtaA.

**FIG 3:**
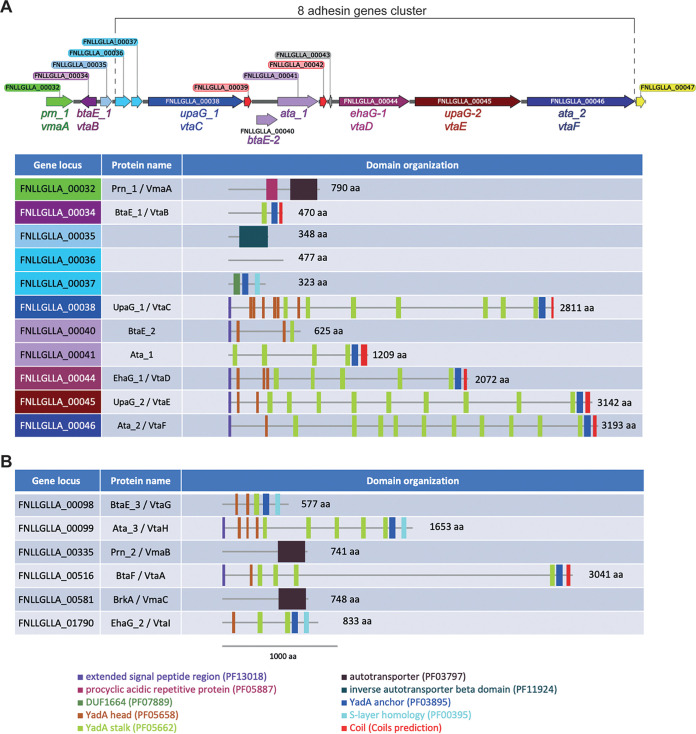
Veillonella parvula autotransporter domain organization. (A) Genetic organization of the V. parvula SKV38 autotransporter adhesin gene cluster and the corresponding adhesin domain organization. (B) Domain organization of the six remaining V. parvula SKV38 autotransporter adhesins encoded by genes located outside the cluster. Domains were detected with the HMMER package (59); only the domains with E values lower than 10*^−^*^3^ are shown. The presence of a C-terminal coil structure was determined using the COILS program (https://embnet.vital-it.ch/software/COILS_form.html). All V. parvula trimeric ATs display an additional C-terminal domain (an SLH or a coiled-coil domain) following the YadA anchor domain compared to classical trimeric autotransporters. aa, amino acids.

The strong biofilm phenotype displayed by the *ΔvtaA* mutant in a microfermentor led us to suspect that additional adhesins could modulate V. parvula biofilm formation capacity. Indeed, searching the V. parvula SKV38 genome revealed multiple genes encoding autotransporters (Table 1): three Va classical monomeric autotransporters with a characteristic PFAM_PF03797 autotransporter β domain (renamed *Veillonella* monomeric autotransporters A to C [VmaA to -C]) and eight other putative Vc trimeric autotransporters with a characteristic PFAM_PF03895 YadA anchor domain (renamed *Veillonella* trimeric autotransporters B to I [VtaB to -I]). We also identified several partial autotransporters: FNLLGLLA_00035, which contains only a PFAM_PF11924 Ve inverse autotransporter β domain but no putative α domain that normally carries the function of the protein, and FNLLGLLA_00036-37 and FNLLGLLA_00040-41, which are homologs of V. parvula DSM2008 Vpar_0041 and Vpar_0048, respectively, and appear to be split in SKV38 (Table 1). Interestingly, domain analysis of all trimeric ATs of V. parvula SKV38 showed that they possess an extra C-terminal domain (S-layer homology [SLH] or coiled-coil domain) after the YadA anchor domain that is not found in classical trimeric ATs. Among those, six autotransporter genes plus *FNLLGLLA_00035*, *FNLLGLLA_00036-37*, and *FNLLGLLA_00040-41* form a potential genomic cluster coding for adhesins (Fig. 3A), whereas the six others are located in different areas of the genome (Fig. 3B).

**TABLE 1:** V. parvula SKV38 autotransporters

Locus tag	PROKKA gene name	Genome position		Gene size (kb)	Strand	Description	DSM2008 homolog	Name	Class
		Start	End						
FNLLGLLA_00032	*prn 1*	39354	41723	2.370	Forward	Autotransporter	Fusion Vpar_0036-0037	VmaA	Va
FNLLGLLA_00034	*btaE 1*	42345	43754	1.410	Reverse	Trimeric autotransporter, YadA like	Vpar_0039	VtaB	Vc
FNLLGLLA_00035	Hypothetical protein	44146	45189	1.040	Forward	Autotransporter (partial)	Vpar_0040		Ve
FNLLGLLA_00036	Hypothetical protein	45453	46883	1.431	Forward	None	Split Vpar_0041		?
FNLLGLLA_00037	*omp-alpha*	46910	47878	0.969	Forward	Trimeric autotransporter/S-layer homology domain	Split Vpar_0041		Vc?
FNLLGLLA_00038	*upaG 1*	48397	56829	8.433	Forward	Trimeric autotransporter, YadA like	Vpar_0042	VtaC	Vc
FNLLGLLA_00040	*btaE 2*	57966	59840	1.875	Forward	Trimeric autotransporter, YadA like (partial)	Split Vpar_0048		?
FNLLGLLA_00041	*ata 1*	59837	63463	3.627	Forward	Trimeric autotransporter, YadA like	Split Vpar_0048		Vc?
FNLLGLLA_00044	*ehaG 1*	65300	71515	6.216	Forward	Trimeric autotransporter, YadA like	Vpar_0051	VtaD	Vc
FNLLGLLA_00045	*upaG 2*	71995	81420	9.426	Forward	Trimeric autotransporter, YadA like	Vpar_0052	VtaE	Vc
FNLLGLLA_00046	*ata 2*	81941	91519	9.579	Forward	Trimeric autotransporter, YadA like	Vpar_0053	VtaF	Vc
FNLLGLLA_00098	*btaE 3*	151792	153522	1.731	Forward	Trimeric autotransporter/S-layer homology domain	Vpar_0100	VtaG	Vc
FNLLGLLA_00099	*ata 3*	154024	158982	4.959	Forward	Trimeric autotransporter/S-layer homology domain	Absent	VtaH	Vc
FNLLGLLA_00335	*prn 2*	414666	416888	2.223	Forward	Autotransporter	Vpar_0330	VmaB	Va
FNLLGLLA_00516	*btaF*	581236	590358	9.123	Forward	Trimeric autotransporter, YadA like	Vpar_0464	VtaA	Vc
FNLLGLLA_00581	*brkA*	668340	670583	2.244	Forward	Autotransporter	Vpar_1322	VmaC	Va
FNLLGLLA_01790	*ehaG 2*	1943661	1946159	2.499	Reverse	Trimeric autotransporter/S-layer homology domain	Vpar_1664	VtaI	Vc

**FIG 4:**
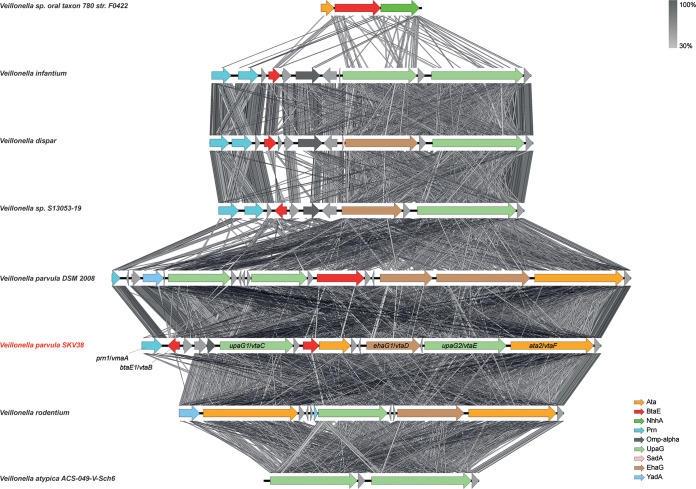
Synteny of the adhesin gene cluster in a selection of *Veillonella* species. The synteny of the proteins of the cluster between the closest relatives was assessed using EasyFig (66). Oblique lines between genes represent tblastx identities (program parameters: maximum E value of 10^12^, minimum length of 30, minimum identity of 30). The V. parvula SKV38 strain used in this study is presented in red. The annotation of the genes of the cluster is indicated on the right.

We selected eight *Veillonella* strains, including SKV38 and DSM2008, to study more precisely the evolution of the adhesin cluster. The trimeric autotransporter adhesins seem to evolve dynamically with numerous domain swaps, duplications, and reductions of gene copies, likely through homologous recombination, suggesting rapid evolutionary changes in the repertoire of *Veillonella* adhesins (Fig. 4). Duplications and deletions could be eased by the presence of short ORFs annotated as hypothetical proteins presenting a high degree of sequence identity. The most basal strain in the *Veillonella* phylogeny has a minimal cluster of only three adhesin genes. Throughout the *Veillonella* genus, the size of the cluster is very variable, with a minimal form in V. atypica, with only two adhesins. This specific adhesin locus, immediately upstream of rRNA-coding genes, is to our knowledge a peculiar genomic character of the *Veillonella* genus and is not found in other genera of the *Veillonellaceae*, suggesting that it originated in the common ancestor of all *Veillonella* species.

### The cluster of trimeric autotransporters is involved in surface binding and not aggregation.

**FIG 5:**
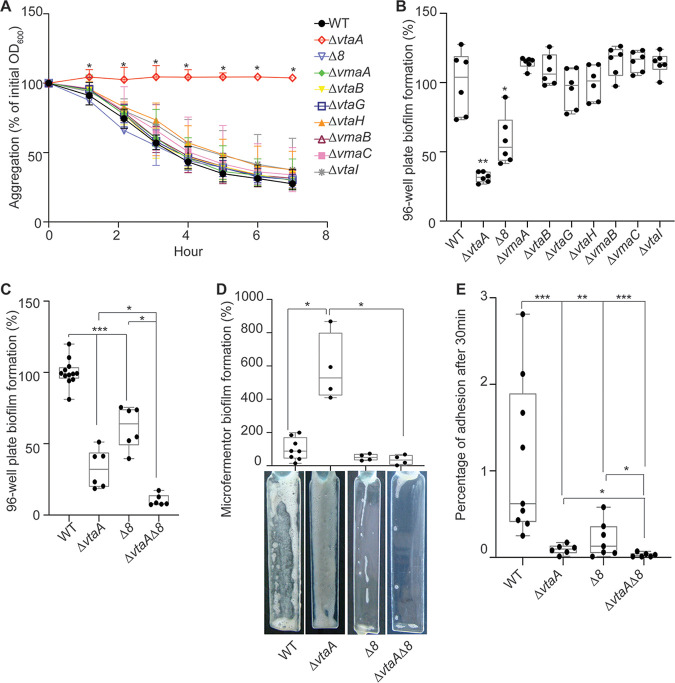
A cluster of eight trimeric autotransporters is involved in surface binding. (A) Aggregation curve in a spectrophotometry cuvette. A value of 100% represents lack of aggregation, and 0% represents complete sedimentation of the culture. Medians of 6 biological replicates are shown, and error bars represent 95% confidence interval. *, significant by Mann-Whitney test, corrected for multiple testing with Bonferroni correction; significance is achieved if the *P* value is <0.007. (B and C) Results of 96-well plate biofilm assay after 24 h of growth in BHILC. The mean for the WT is adjusted to 100%. Min-max box plots of 6 biological replicates for each strain are shown; each replicate is the mean of two technical replicates. For panel B, we applied a Mann-Whitney test: *, *P* < 0.05; **, *P* < 0.005. For panel C, we applied the Bonferroni correction for multiple testing, and tests were deemed significant only if the *P* value was <0.01: *, *P* < 0.01; **, *P* < 0.001; ***, *P* < 0.0001. (D) Biofilm formation in a continuous-flow microfermentor on a glass spatula during 48 h in BHILC. The value for the WT was adjusted to 100%. Min-max boxplots of 4 biological replicates for each strain are shown. *, *P* < 0.05 (Mann-Whitney test). A picture of the spatula before resuspension is shown for each mutant below the box plot. (E) Initial adhesion on a glass spatula. The percentage of CFU that adhered to the spatula, controlled by the number of CFU of the inoculation solution, is shown. Min-max box plots of 6 to 9 replicates per strain are represented. *, *P* < 0.05; **, *P* < 0.005; ***, *P* < 0.0005 (Mann-Whitney test).

To assess the function of the potential adhesins identified in the V. parvula SKV38 genome, we constructed, within the cluster of adhesin genes, independent deletion mutants for the two first autotransporters (*vmaA* and *vtaB*) and a large deletion for the eight adjacent genes encoding trimeric autotransporters or partial trimeric autotransporters, here called *Δ8* (*Δ*[*FNLLGLLA_00036* to *vtaF*]). We also generated independent individual mutants for each of the six additional autotransporters located outside the cluster. These mutants were all tested for biofilm formation in 96-well polystyrene plates and for aggregation capacities. None of the mutants, with the exception of the previously mentioned *ΔvtaA* strain, was affected for aggregation capacities (Fig. 5A). The Δ*8* mutant was the sole mutant, in addition to the Δ*vtaA* mutant, to display lower biofilm formation in 96-well polystyrene microtiter plates (Fig. 5B and C), suggesting that the adhesins of this cluster could be involved in biofilm formation independently of cell-to-cell interactions. When tested in a microfermentor, the Δ*8* mutant displayed a slightly reduced ability to form mature biofilm, but this was not statistically different from that of the WT (Fig. 5D). This reduced ability to form mature biofilms was actually more visible when observing SEM images, since the *Δ8* mutant only poorly covered the coverslip, with sporadic aggregates of cells producing extracellular matrix (Fig. S4). An initial assay of adhesion to a glass spatula showed that both the Δ*vtaA* and Δ*8* strains displayed a lower percentage of initial adhesion than the WT, suggesting that VtaA-mediated autoaggregation contributed to initial adhesion of the WT strain while the adhesin cluster is probably directly involved in surface binding (Fig. 5E). This also indicates that the *ΔvtaA* strain does not adhere to glass better than the WT, and so the increased biofilm formation of the *Δvta* strain in a microfermentor arises during the continuous-flow culture step. The effect of deleting *vtaA* and the 8 adhesin genes on initial adhesion was additive, since a *ΔvtaA Δ8* double mutant showed a reduced initial adhesion on the microfermentor spatula compared to that of either the WT, *ΔvtaA*, or *Δ8* strain (Fig. 5E). In addition, the *ΔvtaA Δ8* mutant formed 17 times less biofilm than the *ΔvtaA* mutant in the microfermentor, indicating that in the absence of VtaA, the adhesins encoded by some of these eight genes strongly promote mature biofilm formation in the microfermentor (Fig. 5D).

Taken together, these results demonstrate the differential contribution of VtaA and part of the adhesin cluster to V. parvula SKV38 adhesion and highlight the existence of potential interference mechanisms between them.

### *FNLLGLLA_01127* encodes an HD phosphatase that inhibits biofilm formation.

**FIG 6:**
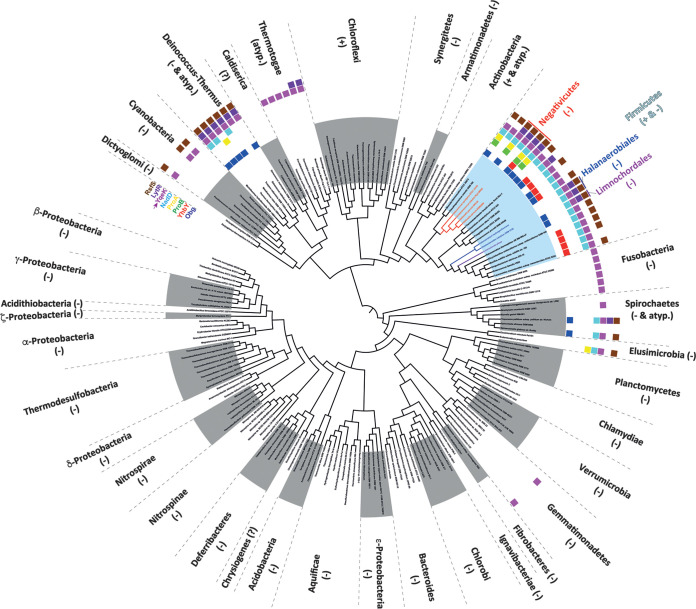
Occurrence and synteny of HD phosphatase (YqeK) in diderm and monoderm bacteria. The presence of the cluster was investigated using MacSyFinder (61), and the results were plotted onto a schematic reference tree of 187 cultivable bacteria among the 390 of the analyzed data bank. The cell wall status of each phylum is indicated as follows: −, diderm with LPS, +. monoderm; atyp., diderm without LPS; ?, unclear. For the *Firmicutes*, the diderm lineages are indicated in red (*Negativicutes*), blue (*Halanaerobiales*), and purple (*Limnochordales*).

**FIG 7:**
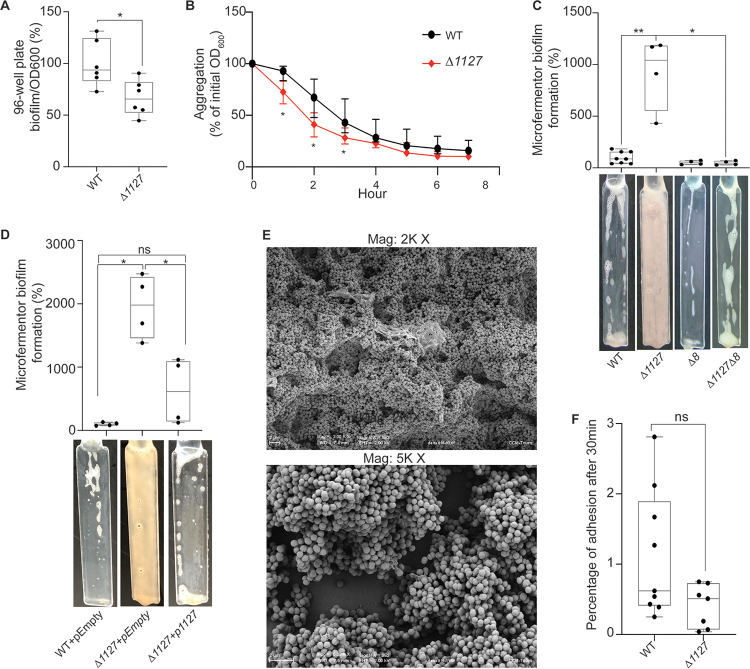
FNLLGLLA_01127 represses biofilm formation in a microfermentor. (A) Results of 96-well plate biofilm assay after 24 h of growth in BHLC corrected by optical density at 600 nm (0D_600_) after 24 h growth in plate. The mean value for the WT is adjusted to 100%. Min-max box plots of 6 biological replicates for each strain are shown, and each replicate is the mean of two technical replicates. *, *P* < 0.05 (Mann-Whitney test). (B) Aggregation curve in a spectrophotometry cuvette. A value of 100% represents lack of aggregation, and 0% represents complete sedimentation of the culture. Medians of 6 biological replicates are shown, and error bars represent 95% confidence interval. *, significant by Mann-Whitney test, corrected for multiple testing with Bonferroni correction; significance is achieved if the *P* value is <0.007. (C) Biofilm formation in a continuous-flow microfermentor on a glass spatula during 48 h in BHILC. The mean value for the WT is adjusted to 100%. Min-max box plots of 4 biological replicates for each strain are shown. *, *P* < 0.05; **, *P* < 0.005 (Mann-Whitney test). A picture of a spatula before resuspension is shown for each strain below the histogram. (D) Biofilm formation in a continuous-flow microfermentor on a glass spatula during 48 h in BHILC plus chloramphenicol. The mean value for WT+pEmpty is adjusted to 100%. Min-max box plots of 4 biological replicates for each strain are shown. *, *P* < 0.05 (Mann-Whitney test). A picture of a spatula before resuspension is shown for each strain below the boxplot. (E) Scanning electronic microscopy of Δ*1127* biofilm grown under continuous flow of BHILC in a microfermentor on a plastic microscopy slide. Magnifications, ×2,000 and ×5,000. (F) Initial adhesion on glass spatula. The percentage of CFU that adhered to the spatula in 30 min, controlled by the number of CFU of the inoculation solution, is shown. Min-max box plots of 6 to 9 replicates per strain are shown. *, *P* < 0.05 (Mann-Whitney test); ns, not significant.

In addition to genes encoding potential T5SS proteins, we also identified a transposon mutant in *FNLLGLLA_01127*, encoding a protein of the HD phosphatase superfamily (Fig. 1B). The *FNLLGLLA_01127* gene product is homologous to YqeK, a putative phosphatase required for pellicle formation and the development of biofilm in B. subtilis (18). *FNLLGLLA_01127/yqeK* is found in a cluster of genes (*obg*, *yhbY*, *proB*, *proA*, *nadD*, *yqeK*, *lytR*, and *rsfS*) whose synteny is very well conserved among *Negativicutes*. This cluster, or part of it, is also well conserved in almost all *Firmicutes* genomes we analyzed, both monoderm and diderm, as well as in members of other diderm phyla phylogenetically close to the *Firmicutes*, notably *Deinococcus-Thermus* (Fig. 6; see Fig. S5 and Data Set S2 in the supplemental material). A *FNLLGLLA_01127* deletion mutant (*Δ1127* mutant) had a lower carrying capacity than the WT, perhaps due to higher mortality during the stationary phase (Fig. S1), and a moderate 1.5-fold decrease in biofilm formation in microtiter plates after correcting for the growth defect (Fig. 7A). This mutant also displayed a slightly higher aggregation rate than the WT at early time points (Fig. 7B). The strongest phenotype of this mutant was detected in the microfermentor, with a 9-fold increase in biofilm formation compared to that of the WT (Fig. 7C). Expression of the *FNLLGLLA_01127* gene in *trans* (plasmid p1127) did not complement the observed growth defect (Fig. S1B), but it did complement the increased biofilm formation in the microfermentor (Fig. 7D), showing that deletion of *FNLLGLLA_01127* might have had polar effects on downstream genes of the operon, causing a growth defect, but that *FNLLGLLA_01127* alone was responsible for the observed inhibition of biofilm formation. Scanning electronic microscopy showed that the *Δ1127* mutant, similarly to the *ΔvtaA* mutant, formed a thick layered biofilm, although with fewer filaments and protein deposits than the WT (Fig. 7E). However, in contrast to the *ΔvtaA* or *Δ8* mutant, the *Δ1127* mutant showed no defect in initial adhesion to a glass spatula (Fig. 7F). Interestingly, a *Δ1127 Δ8* double mutant formed almost 20 times less biofilm than the *Δ1127* mutant in the microfermentor (Fig. 7C), suggesting that at least some of the autotransporters of the cluster were necessary for the observed strong biofilm formation by the *Δ1127* mutant in the microfermentor.

## DISCUSSION

Originally described as a social organism mostly living in biofilm communities (8), *Veillonella* is a known bacterial member of multiple human microbiota. Biofilm formation and adhesion are important in these niches, but their study in *Veillonella* has been hindered by the lack of efficient genetic tools. Here, we used genetic tools adapted from *Clostridia* to characterize factors promoting biofilm formation in a naturally competent Veillonella parvula isolate.

We identified a T5SS type Vc trimeric autotransporter, FNLLGLLA_0516 (VtaA), as an important biofilm factor promoting V. parvula SKV38 autoaggregation. In addition to Hag1, a YadA-like autotransporter identified from the related species V. atypica involved in interspecies interactions (22), VtaA represents the second *Veillonella* protein described which is involved in adhesion and the first involved in abiotic surface adhesion and autoaggregation in diderm firmicutes. Beyond the potential impact on *Veillonella* niche colonization, aggregation capacity is known to contribute to bacterial protection from environmental stresses or host responses (23), promotion of host colonization (24), or pathogenesis (25) in various bacterial species. VtaA is homologous to the Brucella suis trimeric autotransporter BtaF. However, while B. suis BtaF promotes biofilm formation *in vitro*, it was not shown to promote aggregation (21), suggesting that these two proteins have different functions.

In diderm bacteria such as Escherichia coli, self-associating autotransporters (SAATs) from the type Va family and type Vc trimeric autotransporters were shown to contribute to biofilm formation through their self-recognition properties (26–32). However, in V. parvula, VtaA-mediated autoaggregation either promoted (on plastic surfaces and under static conditions) or strongly impaired (on glass surfaces and under continuous-flow conditions) biofilm formation, depending on the model used. The *ΔvtaA* mutant initially adhered less to the glass spatula than the WT, even though later it formed much more biofilm; thus, we suspect that the material (glass versus plastic) is not responsible for the observed difference between our two systems. We hypothesize instead that in the WT, VtaA-mediated aggregates are more sensitive to flow than individual cells and are thus washed out of the microfermentor faster and that adhesion to surfaces or to the biofilm extracellular matrix is more important than cell-to-cell interactions when the culture is performed under continuous flow.

Interference between cell surface structures is a well-described mechanism by which bacteria modulate their adhesion properties. In E. coli, multiple structures, such as chaperone-usher fimbriae, lipopolysaccharide (LPS) O antigen, or capsules, interfere with the self-recognizing autotransporter Ag43 though unknown mechanisms (33–36). Therefore, it is possible that in V. parvula, VtaA could compete with other adhesins through steric hindrance or competition for membrane export and thus limit biofilm formation under continuous-flow conditions. Consistently, the enhanced biofilm formation of the *ΔvtaA* mutant in the microfermentor was dependent on the presence of eight genes of the cluster of trimeric autotransporters, suggesting a competition between VtaA and an adhesin(s) of this cluster. Moreover, we noticed that both VtaA and the 8-gene cluster are necessary for full initial adhesion to a glass spatula in an independent manner, suggesting that any competition between them arises only later on, during continuous-flow cultures. Understanding the exact contributions of these different trimeric autotransporters to biofilm formation and their interplay with VtaA will require further characterization.

Analysis of the V. parvula SKV38 genome revealed the presence of seven other potential full-length autotransporters but no other types of classical diderm adhesins. None of them appeared to be involved in cell-to-cell interactions or biofilm formation on abiotic surfaces, and their function remains to be fully elucidated. As V. parvula is present in different microbiota, it is expected that a large arsenal of adhesion factors is necessary to adhere under different mechanical constraints and on different surfaces, such as tooth enamel or various epithelia. Moreover, *Veillonella* is known to coaggregate with streptococci (37–39), which produce the favored *Veillonella* carbon source, lactate (8), and it was shown to specifically coaggregate with *Streptococcus* and *Actinomyces* strains from the same microbiota, showing that coaggregation could have strong implications for niche colonization of these bacteria (40). V. parvula and other *Veillonella* spp. are also associated with different opportunistic infections, and the contribution of their adhesins to pathogenicity remains to be addressed. Finally, some autotransporters have been shown to carry nonadhesive functions, including protease activity (41), but we detected no classical protease domain in the *Veillonella* autotransporters.

Trimeric autotransporters possess a characteristic YadA anchor domain (PF03895) that is found mainly in *Proteobacteria* but also in *Cyanobacteria*, *Verrumicrobia*, *Planctomycetes*, *Kiritimatiellaeota*, *Chlorobi*, *Synergistetes*, *Fusobacteria*, and *Negativicutes* (https://pfam.xfam.org/family/PF03895 [December 2019]) (42). Interestingly, the YadA anchor of V. parvula SKV38 and all *Veillonella* trimeric autotransporters is not at the very end of the C terminus, where it is usually found in *Proteobacteria*, but is before the C terminus, followed by either a coiled domain or a S-layer homology (SLH) domain (Fig. 3; see Data Set S1 in the supplemental material). While the function of the coiled domain is unknown, in some bacteria the periplasmic SLH domain binds to peptidoglycan (43), suggesting that *Veillonella* trimeric autotransporters could be noncovalently attached to the peptidoglycan. These extra domains after the YadA anchor are also found in other *Negativicutes* (notably the extra SLH domain) and in some other diderm phyla phylogenetically related to the *Firmicutes*, such as *Synergistetes* and *Fusobacteria* (Data Set S1). In addition to possessing trimeric autotransporters with an extra coiled C-terminal domain, the fusobacterium Streptobacillus moniliformis ATCC 14647 carries eight genes encoding unique trimeric autotransporters with an extra OmpA family domain (PF00691) at their extreme C termini, a domain known to display affinity to peptidoglycan (44) (Data Set S1). These data suggest that a subset of phylogenetically close diderm bacteria have evolved trimeric autotransporters integrating different peptidoglycan binding domains. Whether these domains have an impact on trimeric autotransporter function or exposure to the surface, or more generally on outer membrane stabilization, is presently unknown.

Our screening also led to the identification of FNLLGLLA_01127, the homolog of B. subtilis YqeK, a putative phosphatase required for pellicle formation and the development of biofilm (18). Staphylococcus aureus YqeK was recently shown to be a nucleosidase hydrolyzing diadenosine tetraphosphate (Ap4A) into ADP (45). In Pseudomonas fluorescens, an increased level of Ap4A increases the cyclic di-GMP (c-di-GMP) concentration and enhances cell surface exposure of the large adhesin LapA, thus inducing biofilm formation (46). c-di-GMP regulates biofilm formation by modulating production of a variety of cell surface appendages or exopolysaccharides in both monoderm and diderm bacteria (50, 51). Interestingly, B. subtilis YqeK induces the *epsA-O* operon, which is involved in the production of biofilm matrix-forming polysaccharides (52). Deletion of V. parvula *FNLLGLLA_01127* led to only a minor decrease in biofilm formation in 96-well plates but to a strong increase in continuous-flow biofilm formation that was dependent on the presence of the cluster of trimeric autotransporters. Further work is needed to determine whether FNLLGLLA_01127 directly impacts production of the adhesins of the cluster or participates to the production/regulation of an unknown exopolysaccharide, which, in contrast to the case for B. subtilis, would interfere with the function or exposure of the adhesins of the cluster rather than favor community development.

In this study, we have shown that classical diderm trimeric autotransporters and a potential nucleotidase, conserved in both monoderms and diderms, are crucial for adhesion between cells and/or to surfaces in the diderm firmicute V. parvula. Our work also underscores the rapid evolution of a diverse arsenal of trimeric autotransporters in the *Veillonella* genus, both in numbers and size, probably by efficient recombination favored by gene clustering, allowing rapid adaptation to changing environments. Taken together, our results suggest a complex interplay at the surface of V. parvula between different cell surface structures that may have coevolved for a long time in these atypical firmicutes. Much remains to be discovered about the regulatory circuits controlling these adhesion factors and their role in diderm firmicute biology.

## MATERIALS AND METHODS

### Genome preparation and sequencing.

V. parvula SKV38 genomic DNA was extracted using the Qiagen genomic tip 20G kit. It was sequenced to 1,500× coverage using PacBio sequencing of one single-molecule real-time (SMRT) cell with no multiplexing using the V2.1 chemistry. Only one SMRT cell was used but with no multiplexing, leading to an unusually large amount of subreads: 3 Gbp, meaning about 1,500× coverage assuming a 2.1-Mbp genome. This yielded 338,310 reads with a mean subread length of 9,080 bp and N50 read length of 13,500 bp. The longest subread length is above 70 kbp. We randomly subsampled the data to avoid misassemblies, keeping only 100,000 subreads, which resulted in a 430× coverage. The genome was then assembled using Canu version 1.8 (53) with the default parameters. In particular, subreads below 1,000 bp were dropped. The error correction steps of the Canu algorithm were not tuned, keeping the parameters that control alignment seed length, read length, overlap length, and error rates to their default values. We obtained one contig of 2.146 Mbp and an additional contig of only 1,972 bp that was abandoned due to lack of supporting data and was removed by the circularization process. The obtained assembled genome closely matched the genome size (2.1422 Mbp) and GC content (38.7%; expected, 38.6%) of the reference V. parvula DSM2008 strain. The resulting assembled genome was polished using Pilon (54), but no correction was required. No gaps or drops of coverage was detected based on sequana_coverage output (55, 56). The completeness of the candidate assembly was assessed to be 98% using the bacterial mode and the bacteria_db9 lineage-specific profile library of BUSCO software (57), while the number of complete duplicated or fragmented BUSCOs remained at 0, indicative of complete assembly. Alignment of all reads showed that only 4% (13,028) remained unmapped, and 80% of their length was below 2 kbp. The remaining reads (2,000 reads) mapped on various species and could not be further assembled. Overall, these analyses indicate that the final genome assembly is complete and of good quality.

### Bioinformatic analyses.

The V. parvula SKV38 genome was annotated using PROKKA (58).

For protein domain visualization, PFAM domains (pfam.xfam.org, Pfam 32.0. [42]) were detected using HMMER (59). Domains with an E value lower than 10^−3^ were kept, and in the case of overlapping domains, the domain having the best E value was kept. The presence of C-terminal coil structures was determined using the COILS program (https://embnet.vital-it.ch/software/COILS_form.html) (60).

The search for HD phosphatase (YqeK) cluster homologs was conducted as follows. A local data bank containing 390 genomes representative of bacterial diversity was mined for the presence of a phosphatase containing the HD domain (PF01966) using HMMSEARCH and the –cut_ga option. Protein sequences were then filtered using alignment, functional annotation, protein domain presence, and phylogeny. Synteny was investigated in the locus around *yqeK* by looking, using MacSyFinder (61), for the presence of at least one of the 7 genes surrounding *yqeK* in V. parvula SKV38, namely, *obg* (containing the GTP1_OBG domain, PF01018), *yhbY* (containing the CRS1_YhbY domain, PF01985), *proB* (containing the AA_kinase domain, PF00696), *proA* (containing the Aldedh domain, PF00171), *nadD* (containing the CTP_transf_like domain, PF01467), *lytR* (containing the LytR_cpsA_psr domain, PF03816), and *rsfS* (containing the RsfS domain, PF02410), with no more than eight other genes separating them. All HMM profiles were downloaded from the PFAM site (pfam.xfam.org). As YqeK homologs are widespread in the *Firmicutes*, another local data bank containing 230 representative *Firmicutes* genomes was queried by the MacSyFinder approach as described above. All trees were visualized with ITOL (62). Details of the results are presented in Data Set S2 in the supplemental material.

### Strains and growth conditions.

Bacterial strains and plasmids are listed in Table 2. V. parvula was grown in either brain heart infusion (BHI) medium (Bacto brain heart infusion; Difco) supplemented with 0.1% l-cysteine and 0.6% sodium dl-lactate (BHILC) or SK medium (10 g liter^−1^ tryptone [Difco], 10 g liter^−1^ yeast extract [Difco], 0.4 g liter^−1^ disodium phosphate, 2 g liter^−1^ sodium chloride, and 10 ml liter^−1^ 60% [wt/vol] sodium dl-lactate; described in reference 17) and incubated at 37°C under anaerobic conditions in anaerobic bags (GENbag anaero; bioMérieux no. 45534) or in a C400M Ruskinn anaerobic-microaerophilic station. Escherichia coli was grown in lysogeny broth (LB) (Corning) medium under aerobic conditions at 37°C. When needed, 20 mg liter^−1^ chloramphenicol (Cm), 200 mg liter^−1^ erythromycin (Ery), or 2.5 mg liter^−1^ tetracycline (Tc) was added to V. parvula cultures, and 100 mg liter^−1^ carbenicillin (Cb) or 5 mg liter^−1^ Tc was added to E. coli cultures. Unless stated otherwise, 100 μg liter^−1^ anhydrotetracycline (aTc) was added to induce the P*_tet_* promoter. All chemicals were purchased from Sigma-Aldrich unless stated otherwise.

**TABLE 2:** Strains and plasmids used in this study

Strain or plasmid	Description	Reference
Veillonella parvula strains		
SKV38	WT	17
9G5	SKV38 *FNLLGLLA_00516*::transposon	This study
5C5	SKV38 *FNLLGLLA_00516*::transposon	This study
5H1	SKV38 *FNLLGLLA_00516*::transposon	This study
3D6	SKV38 *FNLLGLLA_00516*::transposon	This study
7B11	SKV38 *FNLLGLLA_00516*::transposon	This study
2F7	SKV38 *FNLLGLLA_00516*::transposon	This study
3F7	SKV38 *FNLLGLLA_00516*::transposon	This study
5E11	SKV38 *FNLLGLLA_01127*::transposon	This study
Δ*vtaA* mutant	SKV38 *ΔFNLLGLLA_00516*::*tetM*	This study
P*_tet_-vtaA* mutant	SKV38 *catP-Term(fdx)*-P*_tet_-FNLLGLLA_00516*	This study
* Δ8* mutant	SKV38 *ΔFNLLGLLA_00036-46*::*tetM*	This study
* ΔvmaA* mutant	SKV38 *ΔFNLLGLLA_00032*::*tetM*	This study
* ΔvtaB* mutant	SKV38 *ΔFNLLGLLA_00034*::*tetM*	This study
* ΔvtaG* mutant	SKV38 *ΔFNLLGLLA_00098*::*tetM*	This study
* ΔvtaH* mutant	SKV38 *ΔFNLLGLLA_00099*::*tetM*	This study
* ΔvmaB* mutant	SKV38 *ΔFNLLGLLA_00335*::*tetM*	This study
* ΔvmaC* mutant	SKV38 *ΔFNLLGLLA_00581*::*tetM*	This study
* ΔvtaI* mutant	SKV38 *ΔFNLLGLLA_01790*::*tetM*	This study
* ΔvtaA Δ8* mutant	SKV38 *ΔFNLLGLLA_ 00516*::*catP ΔFNLLGLLA_00036-46*::*tetM*	This study
* Δ1127* mutant	SKV38 *ΔFNLLGLLA_01127*::*tetM*	This study
* Δ1127 Δ8* mutant	SKV38 *ΔFNLLGLLA_01127*::*tetM ΔFNLLGLLA_00036-46*::*catP*	This study
WT+pEmpty	SKV38-pBSJL2-*catP*-P*_mdh_*	This study
Δ*1127*+pEmpty mutant	SKV38 Δ*FNLLGLLA_01127*::*tetM*-pBSJL2-*catP*-P*_mdh_*	This study
Δ*1127*+p1127 mutant	SKV38 Δ*FNLLGLLA_01127*::*tetM*-pBSJL2-*catP*-P*_mdh_-FNLLGLLA_01127*	This study
P*_tet_*-ϕ mutant	SKV38-pRPF185*ΔgusA*	This study
P*_tet_-gusA* mutant	SKV38-pRPF185	This study
P_Cwp2_ -*gusA* mutant	SKV38-pRPF144	This study
Plasmids		
pRPF215	mariner Tn delivery plasmid, P*_tet_*::*Himar1* ITR-*ermB*-ITR *catP tetR*	19
pRPF185	Tetracycline-inducible expression system fused with β-glucuronidase *gusA Term*(*fdx*)-P*_tet_-gusA-Term*(*slpA*), *catP*	63
pRPF185*ΔgusA*	pDIA6103, tetracycline-inducible expression system *Term*(*fdx*)-P*_tet_-Term*(*slpA*), *catP*	67
pRPF144	Carries a *Clostridium* constitutive promoter fused with *gusA P_Cwp2_-gusA*	63
pBSJL2	E. coli-*Veillonella* shuttle plasmid, P*_gyrA_*::*tetM*	68
pBSJL2-cat	E. coli-*Veillonella* shuttle plasmid, P*_cat_*::*catP* P*_mdh_* promoter	This study
p1127	pBSJL2-*catP*-P*_mdh_-FNLLGLLA_01127*	This study

### Natural transformation.

Cells were resuspended in 1 ml SK medium adjusted to an optical density at 600 nm (OD_600_) of 0.4 to 0.8, and 10 μl was dotted on SK agar petri dishes. On each drop, 0.5 to 1 μg plasmid or 75 to 200 ng μl^−1^ linear double-stranded DNA (dsDNA) PCR product was added, or water for the negative control. The plates were incubated for 48 h. The biomass was resuspended in 500 μl SK medium, plated on SK agar supplemented with the corresponding antibiotic, and incubated for another 48 h. Colonies were streaked on fresh selective plates, and the correct integration of the construct was confirmed by PCR and sequencing.

### Random mariner transposon mutagenesis.

Plasmid pRPF215, described for *Clostridium* mutagenesis (Addgene 106377) (19), was transformed into V. parvula SKV38 by natural transformation and selected on Cm-supplemented SK agar medium. An overnight culture of V. parvula SKV38-pRPF215 in BHILC was then diluted to an OD_600_ of 0.1 in the same medium, supplemented with aTc, and grown for 5 h to induce the transposase. After induction, the culture was diluted and plated on BHILC supplemented with Ery and aTc for selection and incubated for 48 h. From the resulting colonies, 940 were inoculated in Greiner Bio-one polystyrene flat-bottom 96-well plates (655101), grown in BHILC supplemented with either Ery and aTc or Cm to confirm both the presence of the transposon and the loss of pRPF215, and then kept in 15% glycerol at –80°C. Selected transposon mutants were grown overnight, and the genomic DNA was harvested using the DNeasy blood and tissue kit (Qiagen). The genomic DNA was then sent for whole-genome sequencing at the Mutualized Platform for Microbiology of the Institut Pasteur.

### Cloning-independent allelic exchange mutagenesis.

Site-directed mutagenesis of V. parvula strain SK38 was performed as described by Knapp and colleagues (17). Briefly, 1-kb regions upstream and downstream of the target sequence and the V. atypica tetracycline resistance cassette (*tetM* in pBSJL2) or *catP* resistance cassette from C. difficile (*catP* in pRPF185; Addgene 106367 [63]) were PCR amplified with overlapping primers using Phusion Flash high-fidelity PCR master mix (Thermo Scientific, F548). PCR products were used as templates in a second PCR round using only the external primers, which generated a linear dsDNA with the tetracycline resistance cassette flanked by the upstream and downstream sequences. This construct was transformed into V. parvula by natural transformation, and its integration into the genome was selected by plating on Tc- or Cm-supplemented medium. Positive candidates were further confirmed by a set of PCRs and sequencing around the site. Primers used in this study are listed in Table S1 in the supplemental material.

### Complementation.

We replaced the tetracycline resistance gene and its *gyrA* promoter of the shuttle vector pBSJL2 by a chloramphenicol resistance gene, P*_cat_*::*cat* from pRPF185, by Gibson assembly. Briefly, the inserts and the plasmids were PCR amplified and then mixed with 2× Gibson master mix (100 μl 5× ISO buffer, 0.2 μl 10,000-U/ml T5 exonuclease [NEB number M0363S], 6.25 μl 2,000-U/ml Phusion HF polymerase [NEB number M0530S], 50 μl 40,000-U/ml *Taq* DNA ligase [NEB number M0208S], 87 μl distilled water [dH_2_O]) for 24 reactions and incubated at 50°C for 30 to 60 min.

The resulting plasmid, pBSJL2-cat, was digested by Fastdigest BamHI (Thermo Scientific), and the band obtained was purified from the agarose gel using the QIAquick gel extraction kit (Qiagen) to be used as a linear plasmid in a second Gibson assembly. The genes and the P*_mdh_* promoter of V. parvula SKV38 were amplified by PCR using PhusionFlash master mix and cloned in pBSJL2-cat using Gibson assembly. The mix was then transformed in E. coli DH5α and plated on LB with carbenicillin. The plasmid was harvested using the QIAprep spin miniprep kit (Qiagen) and transformed in V. parvula as described above.

Alternatively, the anhydrotetracycline-inducible expression cassette of pRPF185, here referred to as P*_tet_* (Addgene 106367) (63), was inserted along with a chloramphenicol marker right before the ATG of the target gene, following the procedure described above for cloning-independent allelic exchange mutagenesis. The functionality of P*_tet_* in V. parvula was previously verified using measurement of the aTc-dependent β-glucuronidase activity generated by the presence of pRPF185 transformed in V. parvula SKV38 (see Fig. S3 in the supplemental material).

### Biofilm formation in 96-well microtiter plates.

Overnight cultures in BHILC medium were diluted to an OD_600_ of 0.05 and transferred to three Greiner Bio-one polystyrene flat-bottom 96-well plates, adding 150 μl per well. After 24 h of static incubation, one of the three plates was resuspended by pipetting to measure OD_600_ using a Tecan Infinite-M200-Pro spectrophotometer. The two other plates were used for coloration, as follows. Cultures were removed by carefully pipetting the supernatant out and biofilms fixed with 150 μl Bouin solution (HT10132; Sigma-Aldrich) for 15 min. Bouin solution was removed by inversion, and the biofilms were washed once in water. The biofilms were stained with 150 μl of 1% crystal violet (V5265; Sigma-Aldrich) for 15 min without shaking and then washed in water twice and left to dry. All washes were made by flicking the plate. After drying the plate, crystal violet was dissolved with 200 μl absolute ethanol and transferred to a clean 96-well plate for OD_620_ measurement (Tecan Infinite-M200-Pro spectrophotometer).

### Biofilm formation in microfermentor.

Continuous-flow nonbubbled microfermentors containing a removable spatula were used as described previously (64, 65; https://research.pasteur.fr/en/tool/biofilm-microfermenters/). Briefly, a glass spatula was dipped in an overnight culture diluted to an OD_600_ of 0.5 in 15 ml BHILC for 15 min and returned to the fermentor. Biofilm was grown on the spatula for 48 h at 37°C. BHILC was constantly supplied through a peristaltic pump at 4 rpm. During the last hour, the speed was increased to 10 rpm to remove planktonic bacteria. A mix of filtered 90% nitrogen–5% hydrogen–5% carbon dioxide was also constantly supplied to maintain anaerobic conditions. After 48 h of growth, the spatula was removed, and the biofilm was resuspended by vortexing in 15 ml BHILC. We measured the OD_600_ of the resuspended biofilms with a Smart Spec Plus spectrophotometer (Bio-Rad).

### Aggregation curve.

Overnight cultures were diluted to an OD_600_ of 0.8 in BHI medium in a semimicrospectrophotometry cuvette (Fisherbrand) and left to sediment on the bench in the presence of oxygen, so no growth should occur. The OD_600_ was measured every hour in a single point of the cuvette using a SmartSpec spectrophotometer (Bio-Rad).

### Initial adhesion on glass.

Glass spatulas from microfermentors (described above) were dipped in overnight cultures diluted to an OD_600_ of 0.5 in 15 ml BHI medium for 30 min to let bacteria adhere. The spatulas were washed once in 15 ml BHI by submersion, and the adhering bacteria were resuspended in 15 ml clean BHI by vortexing. The culture used for inoculation, as well as the resuspended bacteria, was serially diluted and plated on an SK agar plate for CFU counting.

### Statistical analysis.

Statistical analysis was performed using either R and Rstudio software or Prism8 (GraphPad Software, Inc.). We used only the nonparametric test and, when applicable, corrected for multiple testing. For microfermentor experiments, 4 replicates of each condition were used. For all the other experiments, at least 6 biological replicates in at least 2 independent experiment were used. A cutoff *P* value of 5% was used for all tests (*, *P* < 0.05; **, *P* < 0.05; ***, *P* < 0.005).

For growth curve analyses, we computed the growth rate and carrying capacity of each biological replicate using the Growthcurver 0.3.0 package in R, and we performed a Mann-Whitney test comparing both parameters for each mutant to those for the corresponding WT.

### Data availability.

The SKV38 annotated genome sequence was deposited in the National Center for Biotechnology Information (NCBI) database under accession number NZ_LR778174.1.

## Supplementary Material

Supplemental file 1

Supplemental file 2

Supplemental file 3

Supplemental file 4

Supplemental file 5

Supplemental file 6

Supplemental file 7

Supplemental file 9

Supplemental file 8

Supplemental file 10
