# Supplementary material for: Autotransporters Drive Biofilm Formation and Autoaggregation in the Diderm Firmicute Veillonella parvula
Source: J Bacteriol. 2020 Oct 8;202(21):e00461-20. doi: 10.1128/JB.00461-20 (PMC7549365; doi:10.1128/JB.00461-20)
Supplement: Supplemental file 1 [file JB.00461-20-s0001.pdf]

**Primer name**	**Sequence**
TetM-F	agtaaaatgcaggcgagtgaag
TetM-R	gtggatccacaggacacaat
TetM-outF	agatttgaattaaagtgtaaaggagga
TetM-outR	tcttgttaacagcgcaattcct
catP-F	ggccttttgctcacatgttc
catP-R	cctgaagttaactatttatcaattcctgc
catP-chF	cgcagtatgtgacggatttc
catP-chR	gcttcctcgctcactgactc
vtaA-extF	ggctgatatgcatcccac
vtaA-extR	cacttacaaggcccacgact
vtaA-5F	gtagctttagccaatgtggc
vtaA-5R	cttcactcgcctgcattttactactttctcctataaactatagtaatattaatgc
vtaA-3F	attgtgtcctgtggatccacgagttgagaactaattttgataatagtc
vtaA-3R	gtatacataccgaaccgctc
cat-vtaA-5R	gaacatgtgagcaaaaggccactttctcctataaactatagtaatattaatgc
cat-vtaF-3F	gataaatagttaacttcagggagttgagaactaattttgataatagtc
0036-extF	cagcctatgcaggggactac
0046-extR	tctgtgccccttcatatggg
0036-5F	gtcagggcggattggagatg
0036-5R	cttcactcgcctgcattttacttttatcctctccattccctaatgaa
0046-3F	attgtgtcctgtggatccactacgttcatactgaacattacgg
0046-3R	tttgcacaatttcacggcc
cat-0036-5R	agaacatgtgagcaaaaggcctttatcctctccattccctaatgaa
cat-0046-3F	gataaatagttaacttcaggtacgttcatactgaacattacgg
vmaC-extF	caatccttgatcgcaacgc
vmaC-extR	ctacgtaatttaacgctcccc
vmaC-3R	tagtgttccggctaggatg
vmaC-3F	attgtgtcctgtggatccaccggcataatttttaattgaatattg
vmaC-5F	atataaaggcgctccttagg
vmaC-5R	cttcactcgcctgcattttactaaaaatctctctttcttacctcgc
vtaH-extF	ggttatggtcactaccataatgc
vtaH-extR	caaaatcaacatatggtcacatcc
vtaH-3R	cctgatctgcgatcagagc
vtaH-3F	attgtgtcctgtggatccaccgcggttaatagaatcgaaag
vtaH-5F	cgcattctaccgccctaac
vtaH-5R	cttcactcgcctgcattttactccggttcataacactcctcc
vtaB-extF	ccgttagacacattttccatgc
vtaB-extR	gagtcccaagtaggtcgtgc
vtaB-3R	cgtatacaattggtgcacgc

vtaB-3F	attgtgtcctgtggatccactaagatccctttatggtagtcctatg
vtaB-5F	accagacacaccgcgatac
vtaB-5R	cttcactcgcctgcattttactctcatcgtatacactcctttgtgtac
vmaA-extF	gcttctttcaattgctattaggc
vmaA-extR	ggtaaaagtacaagtcaacagc
vmaA-3R	gtacaacatgtatctcaagagg
vmaA-3F	attgtgtcctgtggatccacgcgaattattaatagataggagcg
vmaA-5F	tgcatgataataagctattccttc
vmaA-5R	cttcactcgcctgcattttactcataataaccctctcttattcacac
vtaG-extF	caatcccctgatacacgtg
vtaG-extR	catcatcttcagccaccgc
vtaG-3R	cctttgcctgcgcattac
vtaG-3F	attgtgtcctgtggatccacgacgtatacggttcccgtatatac
vtaG-5F	agctattgctgtagggtctc
vtaG-5R	cttcactcgcctgcattttactctctcatcttacaccctcac
vmaB-extF	caagtttcaaggagtactcg
vmaB-extR	gaataaaatattcaaaagcatctcc
vmaB-5F	ttcttcttgggaaatgaagttgactacagt
vmaB-5R	cttcactcgcctgcattttactgttatacctccttaacacttcaac
vmaB_3F	attgtgtcctgtggatccacttaagggtaaaaatagcagaggc
vmaB-3R	gaaaataatggttgagtctcag
vtaI-extF	cccgcatcaaagagcattgg
vtaI-extR	aaacatgcacgatggcccta
vtaI-5F	taataaactagcaggcatgcg
vtaI-5R	cttcactcgcctgcattttactcgtttactattcctccctacatc
vtaI_3F	attgtgtcctgtggatccacttacttgtcatgaattaaccttttaaac
vtaI-3R	caaagagcattggaatgcc
1127-extF	ggtgttcgcattcacggag
1127-extR	gtgcccccattatgttccg
1127-5F	gtagctcaatatatggaaaacac
1127-5R	cttcactcgcctgcattttactcatttattccttcccatatatatggttttc
1127_3F	attgtgtcctgtggatccactggagttttatatggacgaacg
1127-3R	ggtgcaactttcgacatac
pvtaA-3F	gcgttaacagatctgagctccttattactatagtttataggagaaagtatg
pvtaA-3R	ctacagaacgactcgcag
pvtaA-5R	aagaacatgtgagcaaaaggccattcataattctatatagcgcac
pvtaA-extF	ctatctattggggcagg
pvtaA-extR	agcattaacattatatcccgc
pTet-chF	cgttaacagatctgagctcc
pTet-chR	ccttgaattgatcatatgcgg
catR-pTet	tagcttgatgcagaattcgccctgaagttaactatttatcaattcctgc
cat-pTet-F	gataaatagttaacttcagggcgaattctgcatcaagc

pTet-R	aggagctcagatctgttaac
pBSJL2_ΔtetR	tgtattttatgttgttatataaatatg
pBSJL2_ΔtetF	ctgcaggaattcgatatc
cat_pBSJL2_R	catatttatataacaacataaaatacattaactatttatcaattcctgcaattcgt
cat_pBSJL2_F	gatatcgaattcctgcagagtgagctgataccgctcg
pmdh-F	caacaatcactagtggatccataccaaaattcttcaaaaaaatc
pmdh-R	cgttaaaacctcttttcagaaaatatg
mdh-1127-F	tctgaaaagaggttttaacgatgttatcgtttgatgaaatacaac
pBSJL2-1127-R	atattgtgtcctgtggatccttatttacaagattttaatacatcattacg

Table S1- Primers used in this study.
