# Supplementary material for: Autotransporters Drive Biofilm Formation and Autoaggregation in the Diderm Firmicute Veillonella parvula
Source: J Bacteriol. 2020 Oct 8;202(21):e00461-20. doi: 10.1128/JB.00461-20 (PMC7549365; doi:10.1128/JB.00461-20)
Supplement: Supplemental file 2 [file JB.00461-20-s0002.pdf]

SUPPLEMENTAL MATERIAL

Supplemental Material and Methods

Growth curve

Overnight cultures were diluted to 0.05 OD_{600} in 150µL BHILC that had previously been left in anaerobic condition overnight to remove dissolved oxygen, in Greiner flat-bottom 96-well plates. A plastic adhesive film (adhesive sealing sheet, Thermo Scientific, AB0558) was added on top of the plate inside the anaerobic station, and the plates were then incubated in a TECAN Infinite M200 Pro spectrophotometer for 24 hours at 37°C. OD_{600} was measured every 30 minutes, after 900 seconds orbital shaking of 2 mm amplitude.

Scanning electronic microscopy

Spatula carrying Thermanox plastic coverslips (10.5 x 22 mm, Nunc Thermo Scientific) were introduced in our microfermentor system described above. After 48 hours of growth of the biofilm, the microscopy coverslips were submerged in fixation solution: volume/volume mix of glutaraldehyde 4 %/ sodium cacodylate trihydrate buffer 0.2M pH=7.4/ Ruthenium red 0.15 % and kept at 4°C. Scanning electronic microscopy was then performed by the *Laboratoire de biologie cellulaire et microscopie électronique* of the University of Tours, France.

β-glucuronidase assay

Overnight cultures were diluted to OD 0.05 and grown for 2-3 hours after which the inducer aTc was added. Induced cultures and controls were cultured for 3.5 hours and then ß-glucuronidase activity was assessed. 75 µl of the culture was mixed with 675 µl of Z-buffer (60 mM Na_2HPO_4; 40 mM NaH_2PO_4; 10 mM KCl; 1 mM $MgSO_4$; 50 mM HO-CH_2-CH_2-SH), 50 µl of $CHCl_3$ and 25 µl of 0.1% sodium dodecyl sulfate solution, vortexed and incubated 5 min at 37°C. Then 100 µl of 10 mM 4-nitrophenyl-β-D-glucuronide (in water) was added, and the reaction mix was incubated at 37°C until it turned visibly yellow. The reaction was stopped by adding 375 µl of 1M Na2CO3 (in water) and the absorbance at 405 nm wavelength was measured after a centrifugation. Then the β-glucuronidase activity expressed in Miller units was calculated as described previously for β-galactosidase (1).

Light microscopy

Cultures were grown overnight with no shaking and the aggregates at the bottom of the tubes were harvested. 1μL of these aggregates were dropped on multitest 12-well slide (MP Biomedicals glass), covered with a coverslip (24 x 60 mm coverslip, Menzel glaser) and observed using light microscopy, 1000X.

References for Supplemental material

1. Miller J. 1972. Beta-galactosidase assay. Exp Mol Genet 352:355.
