# Supplementary figures and images for: Autotransporters Drive Biofilm Formation and Autoaggregation in the Diderm Firmicute Veillonella parvula
Source: J Bacteriol. 2020 Oct 8;202(21):e00461-20. doi: 10.1128/JB.00461-20 (PMC7549365; doi:10.1128/JB.00461-20)

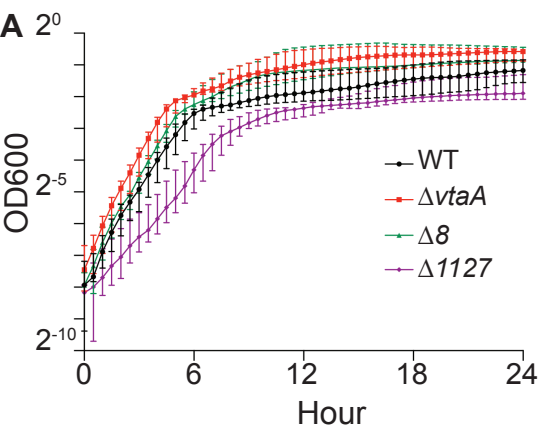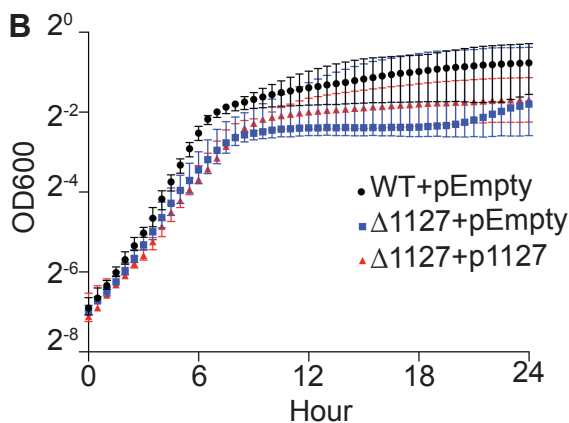

Supplement: Supplemental file 3 [file JB.00461-20-s0003.pdf]

**A**

WT

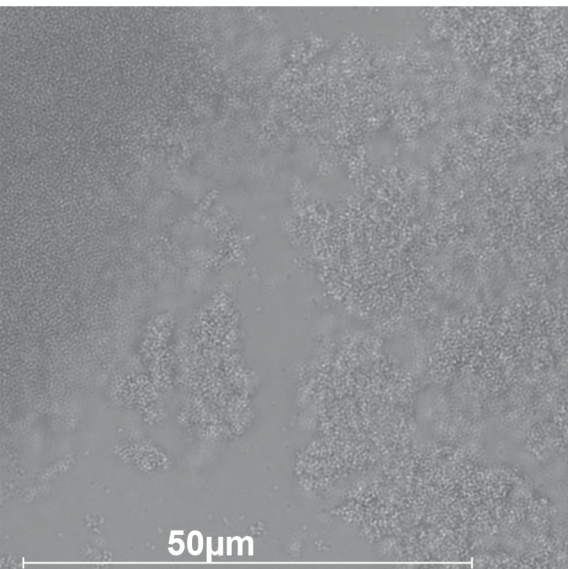 $\Delta vtaA$ 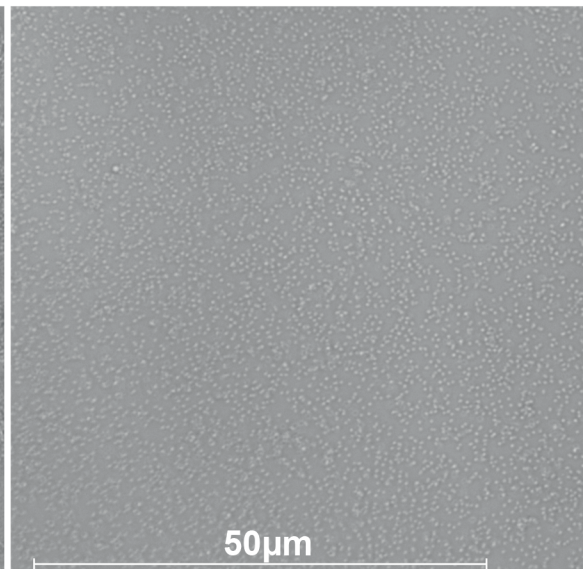**B**

-aTc

+aTc

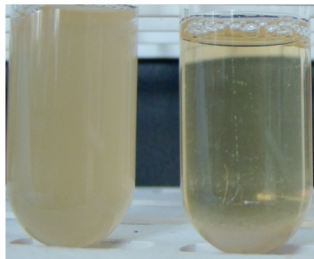

Supplement: Supplemental file 4 [file JB.00461-20-s0004.pdf]

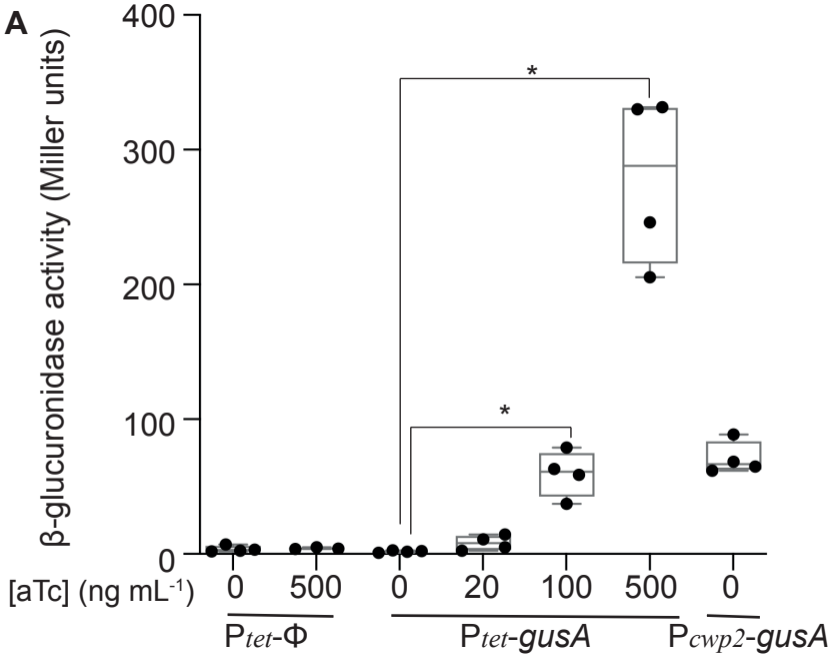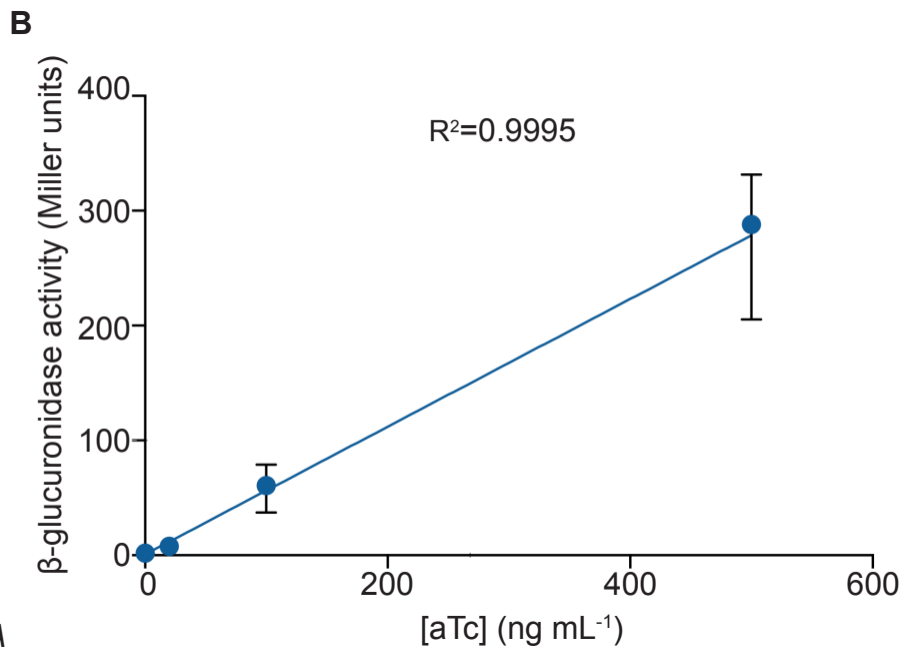

Supplement: Supplemental file 5 [file JB.00461-20-s0005.pdf]

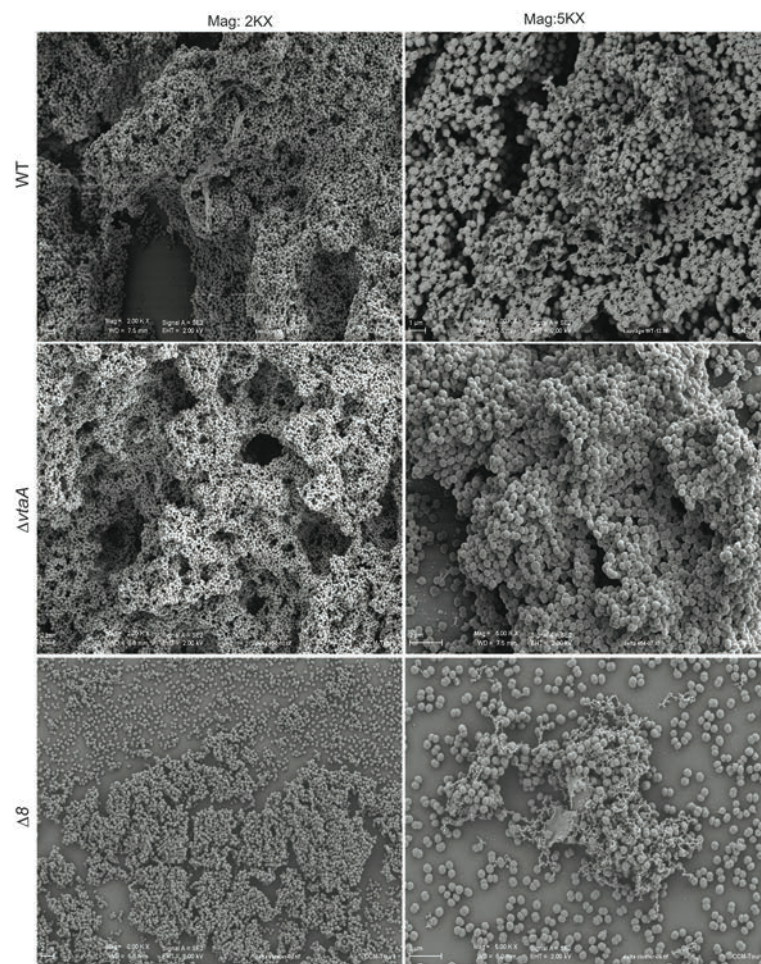

Supplement: Supplemental file 6 [file JB.00461-20-s0006.pdf]

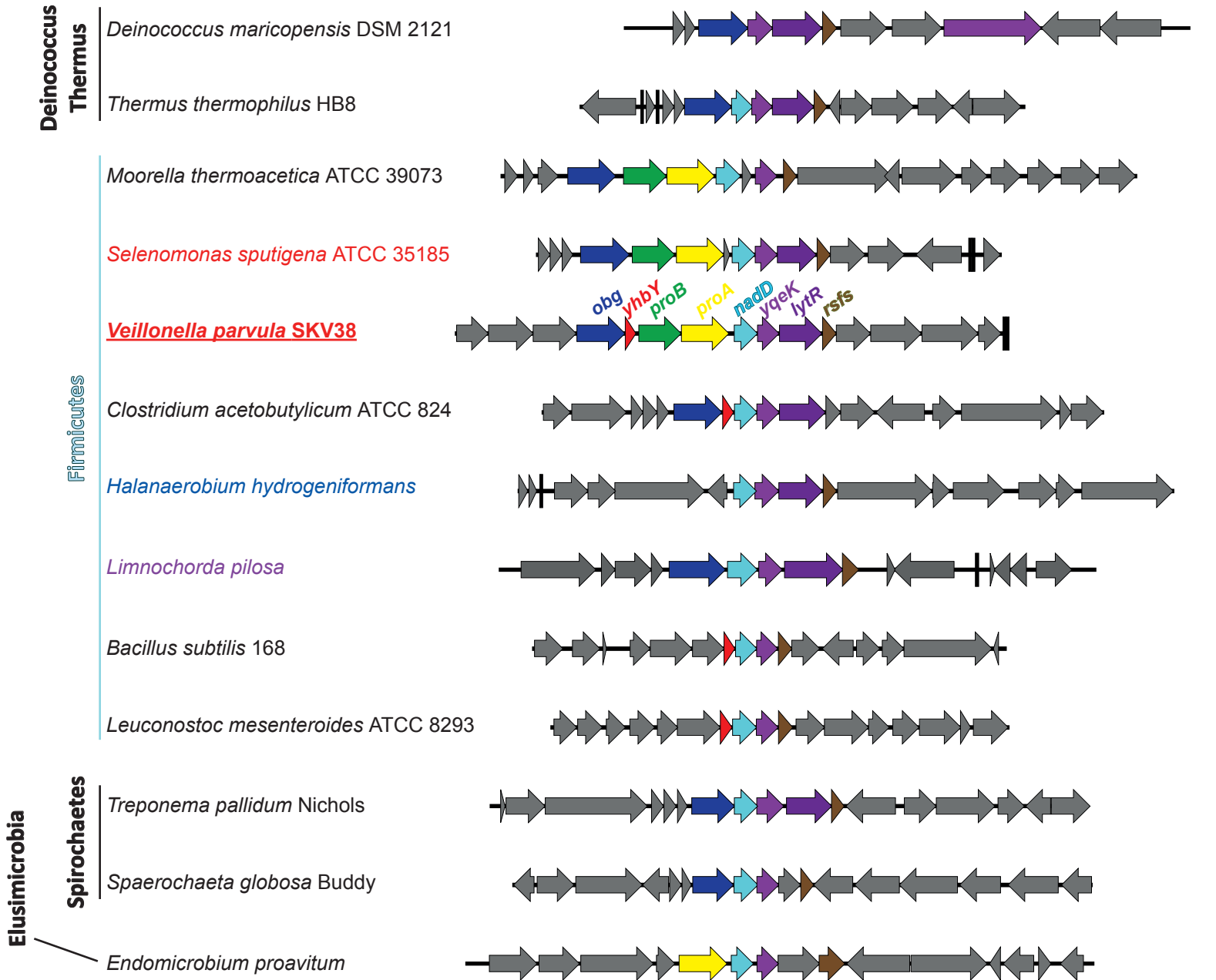

Supplement: Supplemental file 7 [file JB.00461-20-s0007.pdf]
