# Supplementary material for: Autotransporters Drive Biofilm Formation and Autoaggregation in the Diderm Firmicute Veillonella parvula
Source: J Bacteriol. 2020 Oct 8;202(21):e00461-20. doi: 10.1128/JB.00461-20 (PMC7549365; doi:10.1128/JB.00461-20)
Supplement: Supplemental file 10 [file JB.00461-20-so008.pdf]

DESCRIPTION AND LEGEND OF SUPPLEMENTAL MATERIAL

SUPPLEMENTAL FIGURES

Figure S1: **Growth of *V. parvula* mutants.** 24h growth curve in BHILC in 96-well plates. Each point represents the median of 6 biological replicates. Error bars represent 95% confidence interval. For each biological replicate of each strain, the growth rate and the carrying capacity were computed using R library growthcurver. The median of both parameters is indicated in the table below, as well as the p-value of a Mann-Whitney test comparing growth rate and carrying capacity of the mutant with the corresponding WT or WT+pEmpty. **A**. Selected deletion mutants. **B.** Complementation of the Δ1127 mutant. pEmpty correspond to pRPF185. p1127 corresponds to pBSJL2-catP-pmdh-FNLLGLLA_01127. See detailed Supplemental Material and Methods.

Figure S2: **VtaA mediates auto-aggregation. A.** Light microscopy of overnight cultures of WT and Δ*vtaA* grown in BHILC, 1000X. Scale bar represent 50µm. **B.** Overnight cultures of *pTet-vtaA* strain, where *vtaA* is under the control of the inducible pTet promoter, grown in BHILC with or without 0.1µg/mL aTc.

Figure S3. **Tetracycline inducible promoter (P_{tet}) from *Clostridioides difficile* is active in *V. parvula*. A.** β-glucuronidase assay showing the expression of P_{tet} fused to *gusA* under different concentrations of aTc. SKV38 was transformed with pRPF185*ΔgusA* (P_{tet}-φ) as a negative control, pRPF185 carrying the P_{tet}-*gusA* or pRPF144 carrying a copy of *gusA* under a constitutive promoter from Clostridium (P_{Cwp2} -*gusA*). Min-max boxplot of 4 biological replicates. *p-value<0.05, Mann-Whitney test. **B**. ß-glucuronidase activity measured for SKV38-pRPF185 carrying P_{tet}-*gusA* is represented as a function of the inducer aTc concentration. Each dot is the median of 4 biological replicates, error bars represent 95% confidence interval. The coefficient of correlation R^2 is indicated. See detailed Supplemental Material and Methods.

Figure S4**: Scanning electronic microscopy (SEM) of biofilms grown under continuous flow in microfermentor on microscopy slide, magnified 2K or 5K times**. Biofilms of the *V. parvula* WT, Δ*vtaA* and Δ*8* strains were grown for 48h in BHILC medium under anaerobic conditions on Thermanox plastic coverslips. Biofilms were fixed in a glutaraldehyde/cacodylate/ruthenium red solution before scanning electronic microscopy imaging.

Figure S5**: Conservation of the HD Phosphatase encoding gene (*yqeK*) synteny in selected diderm and monoderm bacteria**. Some species from Figure S5 that contain a minimum of three genes in addition to *yqeK* within the cluster were selected, and the corresponding genetic organizations are represented according to their phylogenetic proximity. The different genes are displayed with different colors. The color code is the same as that of Figure S5. *V. parvula* SKV38 strain is underlined. In this strain *yqeK* corresponds to *FNLLGLLA_01127*.

OTHER SUPPLEMENTAL MATERIAL

Table S1**: Primers used in this study.**

DataSet 1**: Description of trimeric autotransporters containing a Cterminal domain downstream of the YadA_anchor domain.** YadA_anchor-SLH: trimeric autotransporters containing a Cterminal SLH domain donwstream of the YadA_anchor domain detected in all bacterial species (only Negativicutes contain such a SLH domain); YadA_anchor-Coil-Negativi: trimeric autotransporters containing a Cterminal coilded domain donwstream of the YadA_anchor domain detected in Negativicutes ; YadA_anchor-Coil-Others: trimeric autotransporters containing a Cterminal coilded domain donwstream of the YadA_anchor domain detected in all bacterial species except in Firmicutes ; YadA_anchor-otherConfig: trimeric autotransporters containing a Cterminal domain different from SLH or coiled domain and downstream of the YadA_anchor detected in all bacterial species except diderm Firmicutes.

DataSet 2**: Presence/absence of the genes contained in the genetic cluster encompassing the HD phosphatase encoding gene *yqeK***. In the sheet MacSyFBACTERIA the analysis has been done on a database of 390 species representative of the Bacteria. In the sheet MacSyFFIRMICUTES the analysis has been done on a database of 230 species representative of the Firmicutes.

Supplemental Material & Methods: **Description of supplemental Material and Methods.**
